# FBXW4 Acts as a Protector of FOLFOX-Based Chemotherapy in Metastatic Colorectal Cancer Identified by Co-Expression Network Analysis

**DOI:** 10.3389/fgene.2020.00113

**Published:** 2020-03-11

**Authors:** Yiyi Zhang, Lijun Sun, Xiaojie Wang, Yanwu Sun, Ying Chen, Meifang Xu, Pan Chi, Xingrong Lu, Zongbin Xu

**Affiliations:** ^1^Department of Colorectal Surgery, Fujian Medical University Union Hospital, Fuzhou, China; ^2^Department of Oncology, The First Affiliated Hospital of Fujian Medical University, Fuzhou, China; ^3^Department of Plastic Surgery, Fuzhou Dermatosis Prevention Hospital, Fuzhou, China; ^4^Department of Pathology, Fujian Medical University Union Hospital, Fuzhou, China

**Keywords:** colorectal cancer, FOLFOX, gene array chip, weighted gene co-expression network analysis, FBXW4

## Abstract

**Background:**

FOLFOX chemotherapy is one of the most commonly used treatments for colorectal cancer (CRC) patients. However, the efficacy and tolerance of FOLFOX therapy varies between patients. The purpose of this study was to explore hub genes associated with primary chemotherapy-resistance and to explore the possible mechanisms involved from non-European patients.

**Method:**

A weighted gene co-expression network was constructed to identify gene modules associated with chemotherapy resistance in mCRC from China.

**Results:**

A Gene Array Chip was used to detect mRNA expression in 11 mCRC patients receiving preoperative FOLFOX chemotherapy. The immune response was associated with chemotherapy-resistance in microarray data. Through the use of WGCNA, we demonstrated that the crucial functions enriched in chemotherapy-resistance modules were cell proliferation, MAPK signaling pathways, and PI3K signaling pathways. Additionally, we identified and validated FBXW4 as a new effective predictor for chemotherapy sensitivity and a prognostic factor for survival of CRC patients by using our own data and GSE69657. Furthermore, a meta-analysis of 15 Gene Expression Omnibus–sourced datasets showed that FBXW4 messenger RNA levels were significantly lower in CRC tissues than in normal colon tissues. An analysis of the data from the R2: Genomics Analysis and Visualization Platform showed that low FBXW4 expression was correlated with a significantly worse event- and relapse-free survival. Gene set enrichment analysis showed that the mechanism of FBXW4-mediated chemotherapy resistance may involve the DNA replication signal pathway and the cell cycle.

**Conclusion:**

FBXW4 is associated with chemotherapy resistance and prognosis of CRC probably by regulating DNA replication signaling pathways and the cell cycle.

## Introduction

Colorectal cancer (CRC) is the third most common cancer and the second leading cause of cancer-related death worldwide (Edwards et al., 2014). Chemotherapy is one of the most commonly used treatments for CRC patients, and is usually combined with surgery, radiotherapy, immunotherapy, and targeted molecular therapy. Oxaliplatin, fluorouracil plus leucovorin (FOLFOX) is a well-established first-line chemotherapy for CRC patients (Benson et al., 2017). However, the efficacy and tolerance of FOLFOX therapy varies among patients. Patients with chemotherapy-resistant CRC could be exposed to chemotherapy toxicities without any therapeutic benefit. Moreover, patients with the primary (also known as *de novo*) FOLFOX-resistant would not get any benefit from the FOLFOX regimen. Therefore, identification of potential biomarkers and therapeutic targets for patients with chemotherapy-resistant CRC is urgently needed.

High-throughput sequencing technology is commonly used to screen and identify differential genes. Conventional molecular biology methodology only identifies variations and functions of genes independently. An effective alternative is weighted gene co-expression network analysis (WGCNA), an unbiased systematic biological approach. WGCNA elucidates the system-level functionality of a transcriptome, determines correlations among genes, and identifies highly correlated gene modules across microarray data. It can also be used to bridge gaps between individual genes and the associations between occurrence and progression of the disease (Zhang and Horvath, 2005; Langfelder and Horvath, 2008; Tian et al., 2017). Additionally, WGCNA facilitates network-based gene screening methods which can be used to identify and screen for key biomarkers associated with clinical traits in various cancers. However, this efficient bioinformatic approach has not yet been adopted to identify network-centric genes associated with chemotherapy-resistant CRC.

In the present study, we explored mRNA expression profiling in primary chemotherapy-resistant patients compared with chemotherapy-sensitive patients from non-European patients. Then, WGCNA was performed to screen relevant hub genes for chemoresistance in the expression profile. Finally, hub genes were verified using testing sets containing patient tissue samples.

## Materials and Methods

### Subjects and Collection

A total of 11 metastatic CRC (mCRC) patients with synchronous liver metastases were enrolled in our study between January 2017 and December 2017 from the Fujian Medical University Union Hospital, China (the clinicopathological details was in the **Supplemental Table 1**). All patients voluntarily provided written informed consent, received neoadjuvant FOLFOX4 chemotherapy, and underwent R0 resection of primary colorectal tumors after neoadjuvant chemotherapy. And the samples were collected at diagnosis and without any treatment before biopsy from colonoscopy. The chemotherapy protocol was as follows: oxaliplatin, 85 mg/m^2^ body surface area (BSA), day 1, was infused intravenously with calcium folinate, 200mg/m^2^ BSA, days 1 and 2, over 2 h. Next, an intravenous bolus injection of 400 mg/m^2^ BSA fluorouracil was given sequentially, followed by a continuous 22 h intravenous infusion on days 1 and 2. The Response Evaluation Criteria in Solid Tumors (RECIST) was used to evaluate the patients' response after completion of six cycles of chemotherapy (Des Guetz et al., 2009; Ren et al., 2009). No patient achieved a complete response (CR), four achieved partial responses (PR) and were included in the experimental group, another four achieved stable disease (SD), and three had disease progression (PD) and were included in the control group.

### RNA Extraction, Quality Control, Labeling and Array Hybridization

Total RNA was extracted from the tissue mentioned previously using Trizol reagents (Invitrogen), according to the manufacturer's protocol. RNA quantity and quality were measured by NanoDrop ND-1000 and RNA integrity was assessed by standard denatured agarose gel electrophoresis. Sample labeling and array hybridization were performed according to the Agilent One-Color Microarray-Based Gene Expression Analysis protocol (Agilent Technology) with minor modifications.

### Data Analysis

The microarray was analysed by Aksomics Inc (Shanghai, China). The steps were briefly described as follows, Agilent Feature Extraction software (version 10.7.3.1) was used to analyze acquired array images. The GeneSpring GX v11.5.1 software package (Agilent Technologies) was used for quantile normalization and subsequent data processing. Differentially expressed mRNAs were identified through Fold Change filtering. Hierarchical Clustering was performed using Agilent Gene Spring GX software (version 11.5.1). The Gene Ontology (GO) functional analysis and Kyoto Encyclopedia of Genes and Genomes (KEGG) pathway analysis were performed using standard enrichment computation method.

### Data Collection and Preprocessing

**Figure 1:**
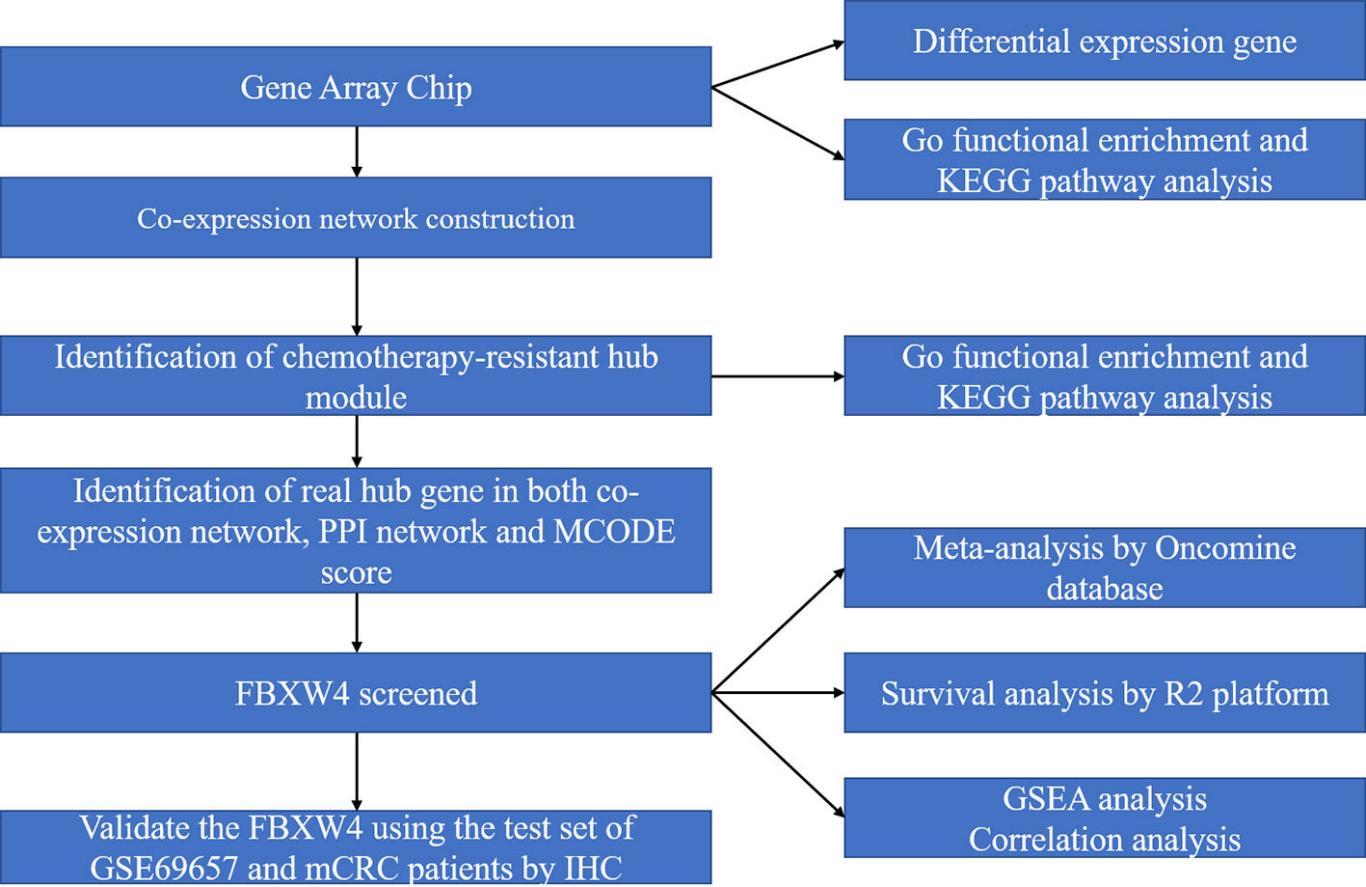
Work flow diagram of data preparation, processing, analysis, and validation in this study. FBXW4, F-box/WD-40; GO, Gene Ontology; GSEA, gene set enrichment analysis; PPI, protein-protein interaction; MCODE, Molecular Complex Detection.

The microarray dataset was retrieved from the Gene Expression Omnibus (GEO) database (https://www.ncbi.nlm.nih.gov/geo/). The microarray data set that was used (GSE69657) came from our previous data submitted by Lu et al (Li et al., 2013). The data set included mRNA expression profiles of 30 advanced CRC patients: 13 patients achieved partial responses and were included in the responder group, six achieved stable disease (SD), and 11 had disease progression (PD) and were included in the non-responder group. This data set (GSE69657) was used as the testing dataset and the validation dataset set to construct co-expression networks. The work flow of this study is shown in **Figure 1**.

### Co-Expression Network Construction

The WGCNA algorithm was described in detail previously (Zhang and Horvath, 2005). Briefly, we first identified the qualification profiles for our data. The co-expression network was constructed using “WGCNA” package in R software. (Horvath and Dong, 2008; Mason et al., 2009). Next, the correlation matrix was established and the soft threshold power was determined by analyzing the network topology. Finally, the topological overlap matrix (TOM) was established (Yip and Horvath, 2007; Botía et al., 2017). Based on the phenotypic data of the groups, we calculated each module p-value using a t-test gene significance.

### Identification of Chemotherapy Sensitivity and Hub Gene

To explore the relevant module, we examined the association between module eigengenes (MEs) and chemotherapy-resistance using Pearson's correlation analyses. To identify hub genes, we first chose the chemotherapy resistance module with the highest correlation coefficient (P < 0.05) in the data set. The module was also the maximum specific weight of all of the modules, and hub genes in the module were defined by module connectivity as measured using the absolute value of the Pearson's correlation. Additionally, a protein-protein interaction (PPI) network was constructed using all genes in the chemotherapy resistance module. A PPI network analysis was performed to screen the hub genes using the Molecular Complex Detection (MCODE) algorithm in Cytoscape software. Finally, we chose the maximum score genes (score = 34) to construct the PPI network to select the “real” hub genes using the maximum degree.

To further verify the hub gene expression in the chemotherapy-resistant and -sensitive group inour data and external validating data set (GSE69657), statistical analyses were performed using SPSSsoftware (ver. 23 SPSS Inc, Chicago, IL) and R software (ver. 3.4.1). The Oncomine database (https://www.oncomine.org) was also used to investigate the differential expression of the hub gene between the CRC and normal groups. Finally, we analyzed the prognostic value of the hub gene on patients with CRC by the R2: Genomics Analysis and Visualization Platform (http://r2.amc.nl). P < 0.05 was considered as the level of significance.

### Gene Set Enrichment Analysis (GSEA) and Immunohistochemical Analysis

To figure out the potential function of FBXW4 in CRC patients, GSEA was performed in patients from our datasets. P < 0.05 and |enrichment score (ES)| > 0.3 were set as the cut-off criteria.

The concentrations of FBXW4 proteins, CD3+ cells, CD4+ cells, and CD8+ cells in the 55 mCRC patients were measured using the immunohistochemical streptavidin-biotin complex method. The characteristics of patients and the criteria was reported in our previous study (Zhang et al., 2018). All analyses were performed in a double-blind manner.

## Results

### Cluster Analysis

**Figure 2:**
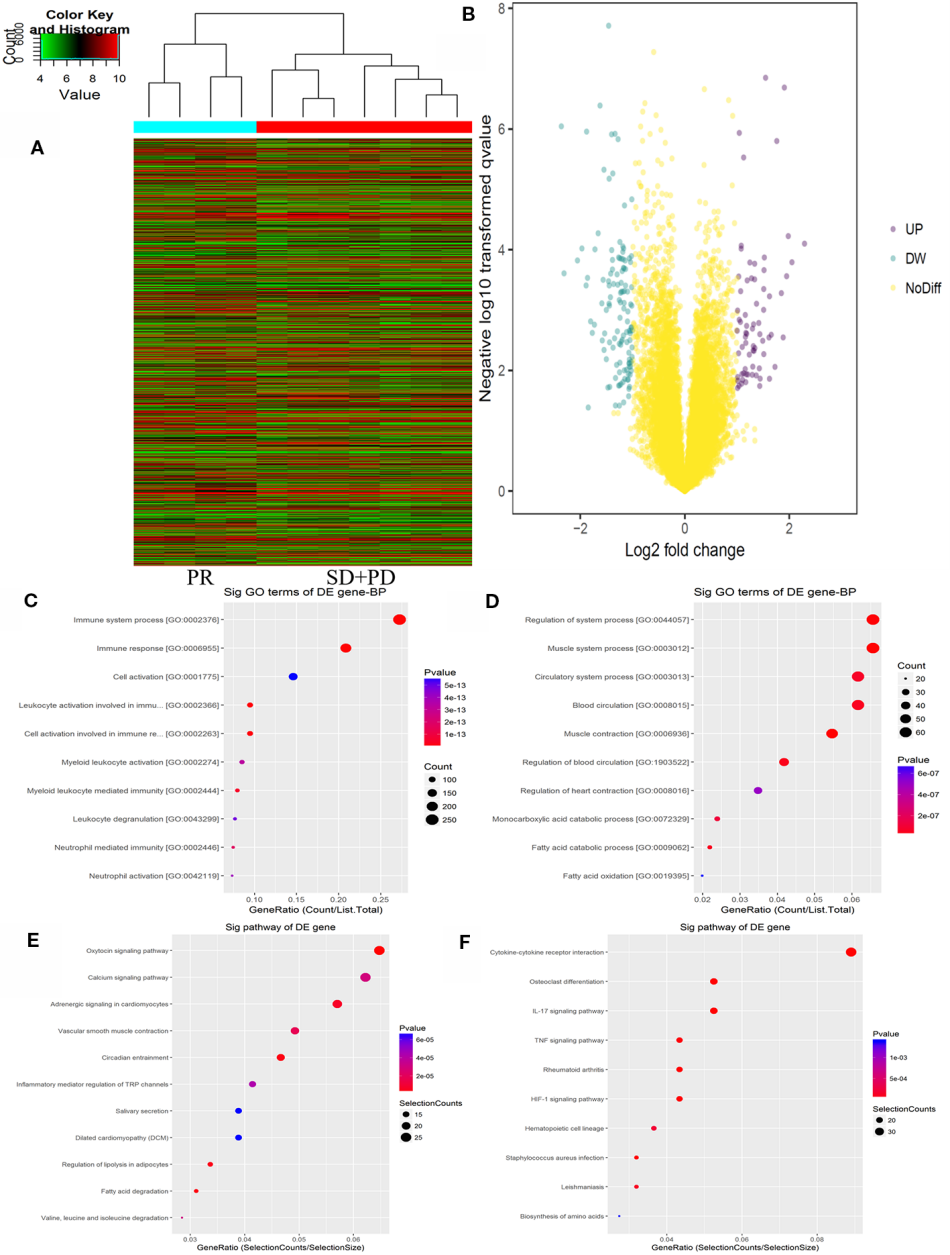
mRNAs expression profile comparison between chemotherapy-resistance and chemotherapy-sensitivity groups. Gene Ontology (GO) functional and Kyoto Encyclopedia of Genes and Genomes (KEGG) pathway analysis of the differentially expressed genes. **(A)** The hierarchical clustering of all targets value of mRNA expression profiling among samples. **(B)** between the chemotherapy-resistance and chemotherapy-sensitivity group. The purple dots indicated the up-regulated genes of mRNAs and the green dots indicated the down-regulated genes of mRNAs. **(C)** GO functional analysis of the top ten functional classifications of the upregulated genes. **(D)** GO functional analysis of the top ten functional classifications of the downregulated genes. **(E)** KEGG pathway analysis of the top ten significant pathways of upregulated genes. **(F)** KEGG pathway analysis of the top 10 pathways of downregulated genes.

The Agilent Gene Chip Array was used to examine gene expression profiles in primary tumor cells. A supervised hierarchical cluster analysis of gene expression profiling data showed that the two groups had a clustering trend (**Figures 2A, B**). The SAM for differentially expressed genes revealed that the two groups significantly differed in tumor cell biology: the expression was significantly upregulated in 17 genes (FDR < 0.01), and downregulated in 50 genes (FDR < 0.01).

### GO Enrichment and KEGG Analysis

GO enrichment analysis was performed to investigate the molecular mechanism of differently expressed genes involved in resistance to the FOLFOX regimen in mCRC patients. We detected the top 10 significant GO terms enriched in both the significantly upregulated and downregulated genes in mCRC patients, respectively (**Figures 2C, D**). The results showed that the top 3 significant GO terms were related to the immune system processes, immune response, and leukocyte activation involved in the immune response in upregulated genes. In the downregulated genes, the top three significant GO terms were related to the muscle system process, muscle contraction, and regulation of the system processes. Moreover, to verify the results of GO enrichment and KEGG analysis, an immunohistochemical analysis was performed to detect tumor infiltrating lymphocytes (TILs) in the mCRC patients. The results demonstrated that CD3+ and CD8+ expression was lower in the chemotherapy resistance group than that in the chemotherapy sensitive group (P = 0.03, P = 0.01). However, the expression difference of CD4+ cells between the chemotherapy resistance group and the chemotherapy sensitive group were similar (P = 0.07). However, the chemotherapy sensitive group had a higher expression of CD4+ cells (**Figures 4G, H**; **Supplemental Figure 2** and **3**). Moreover, by using X-TILE, we determined the optimal cut-off value of the TILs cells. We divided the patients with low or high expression of CD3/4/8+ using the cut-off value and performed K-M analyses. The results demonstrated that high expression of TILs indicated a better prognosis. The above results indicated that high expression of TILs was associated with chemotherapy sensitivity and a better prognosis in mCRC patients receiving FOLFOX chemotherapy (**Supplemental Figure 3**).

Additionally, we analyzed the differential genes in mCRC patients by KEGG analysis (**Figures 2E, F**). The results demonstrated that the top three KEGG pathways were related to the IL-17 signaling pathway, cytokine-cytokine receptor interaction, and the HIF-1 signaling pathway in upregulated genes. The top three downregulated genes were the oxytocin signaling pathway, fatty acid degradation, and circadian entrainment.

### Construction of a Weighted Co-Expression Network and Identification of Key Modules

**Figure 3:**
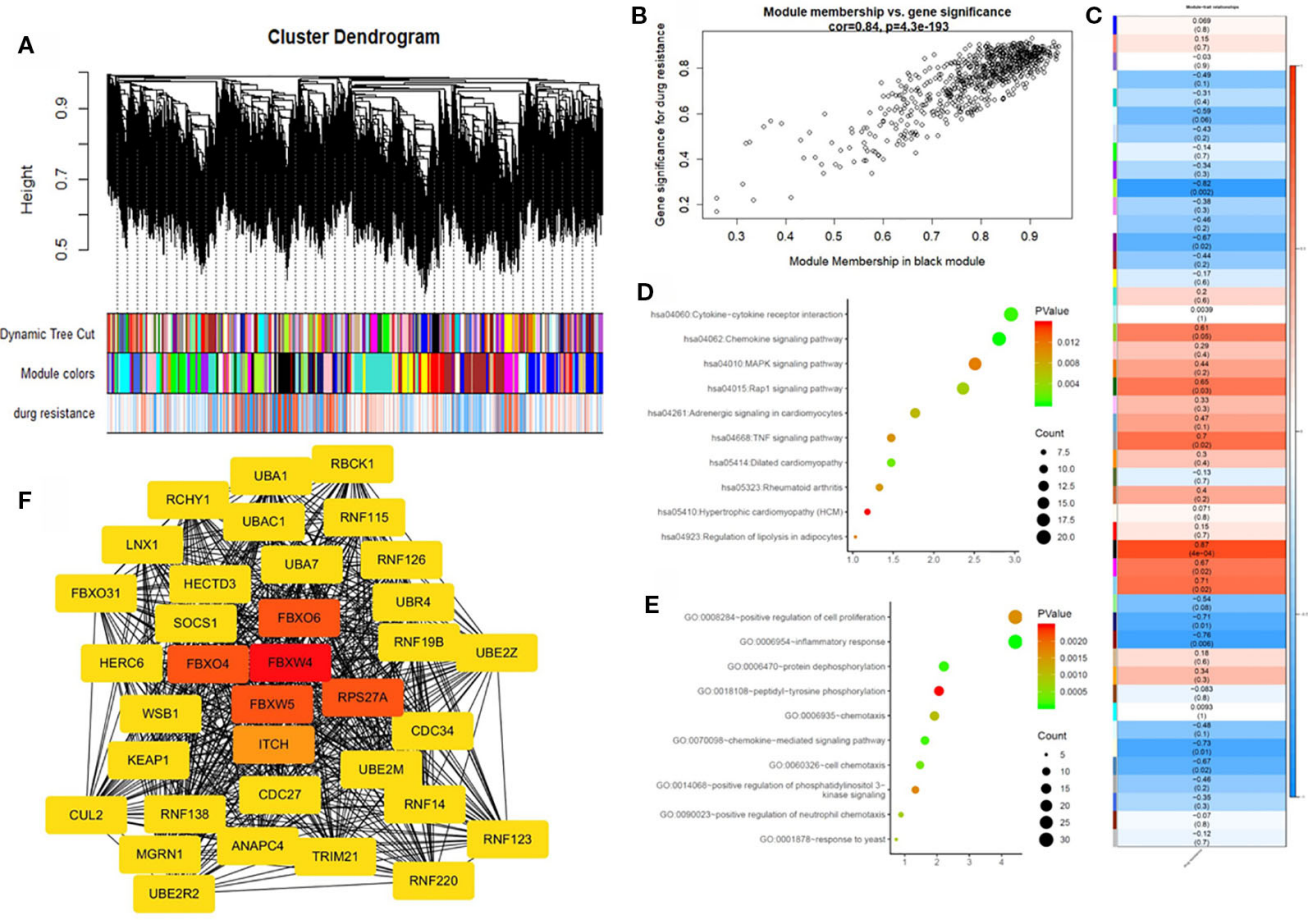
Weighted gene co-expression network analysis (WGCNA) and hub gene screened. **(A)** Dendrogram of all expressed genes in the top 25% of variance clustered based on a dissimilarity measure (1−TOM). **(B)** Scatter plot of the correlation between the black module and chemotherapy resistance. **(C)** Heatmap of the correlation between module eigengenes and chemotherapy resistance. **(D)** KEGG pathway analysis of the top ten pathways of genes in black modules. **(E)** GO functional analysis of the top 10 pathways of genes in black modules. **(F)** Protein-protein interaction network of genes which has the highest score in the MOCDE in the black module. The color intensity in each node was proportional to the degree of connectivity in the weighted gene co-expression network.

To further identify the hub gene, we used the weighted co-expression network to analyze our data (**Figure 3A**). We analyzed the relationship between chemoresistance and 30 modules which were identified by the weighted co-expression network (**Figure 3C**). Among these modules, the ME of the black module had the highest positive correlation with chemoresistance (r = 0.87, P = 0.0004), while the ME of the greenyellow module had the highest negative correlation with chemoresistance (r = −0.82, P = 0.002). Then, we used a Pearson's test to further analyze the relationship between each gene and chemoresistance (**Figure 3B**). We selected the black module as the hub modules for further analysis. The GO functional enrichment analysis was conducted to explore the potential biological functions of the black module. Genes in the black module were primarily enriched for positive regulation of cell proliferation, inflammatory response, and protein dephosphorylation (**Figure 3D**). Additionally, we evaluated the genes in the black modules by KEGG analysis. The results demonstrated that the top three KEGG pathways were related to the chemokine signaling pathway, cytokine-cytokine receptor interaction, and MAPK signaling pathway (**Figure 3E**).

### Hub Gene Identification

By co-expression analysis, 720 genes in the black module were identified as genes with high module connectivity. The genes were then analyzed using the PPI network and 323 genes in the black module were finally chosen as hub genes in the co-expression network. The PPI network was analyzed using the MOCDE algorithm in Cytoscape, We chose the maximum score of 35 genes as the candidate for hub genes in the next analysis. The degree score analysis in Cytoscape was run on the 35 genes (**Figure 3F**). Finally, we chose the most associated gene, FBXW4, as the “real” hub gene.

### Hub Gene Validation

**Figure 4:**
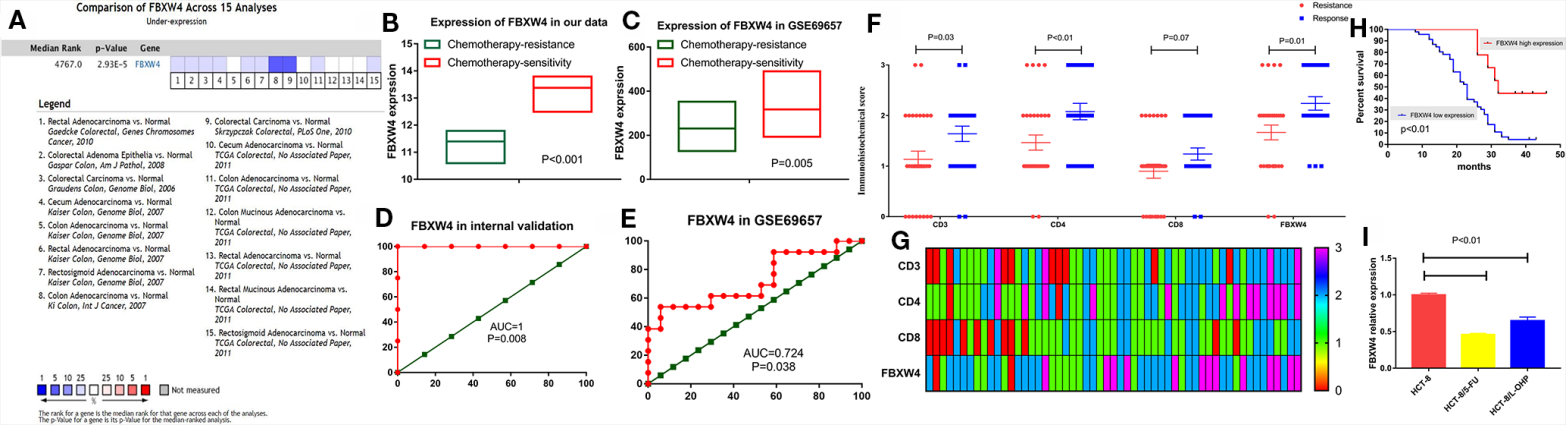
Validation of FBXW4. **(A)** Meta‐analysis of 15 GEO‐sourced data sets mined from the Oncomine database showed that FBXW4 mRNA levels were significantly lower in CRC tissues than in normal colon tissues (P < 0.001). **(B)** In our data (11.40 ± 0.40 vs 13.38 ± 0.60, P = 0.000) and **(C)** GSE69657(231.13 ± 52.16 and 317.35 ± 102.57, P = 0.006). ROC curves and AUC statistics to evaluate the predictive efficiency of the FBXW4 in our data and external data to distinguish chemotherapy-resistance from chemotherapy‐sensitive CRC cases from **(D)** our data and **(E)** GSE69657. The immunohistochemical score of FBXW4, CD3, CD4, and CD8 in FOLFOX-resistance and -sensitive cancerous tissue, **(F)** and **(G)**. The overall survival for mCRC patients stratified by the expression level of FBXW4 **(H)**. The FBXW4 expression in the FOLFOX resistance cell lines and parental cells **(I)**.

**Figure 5:**
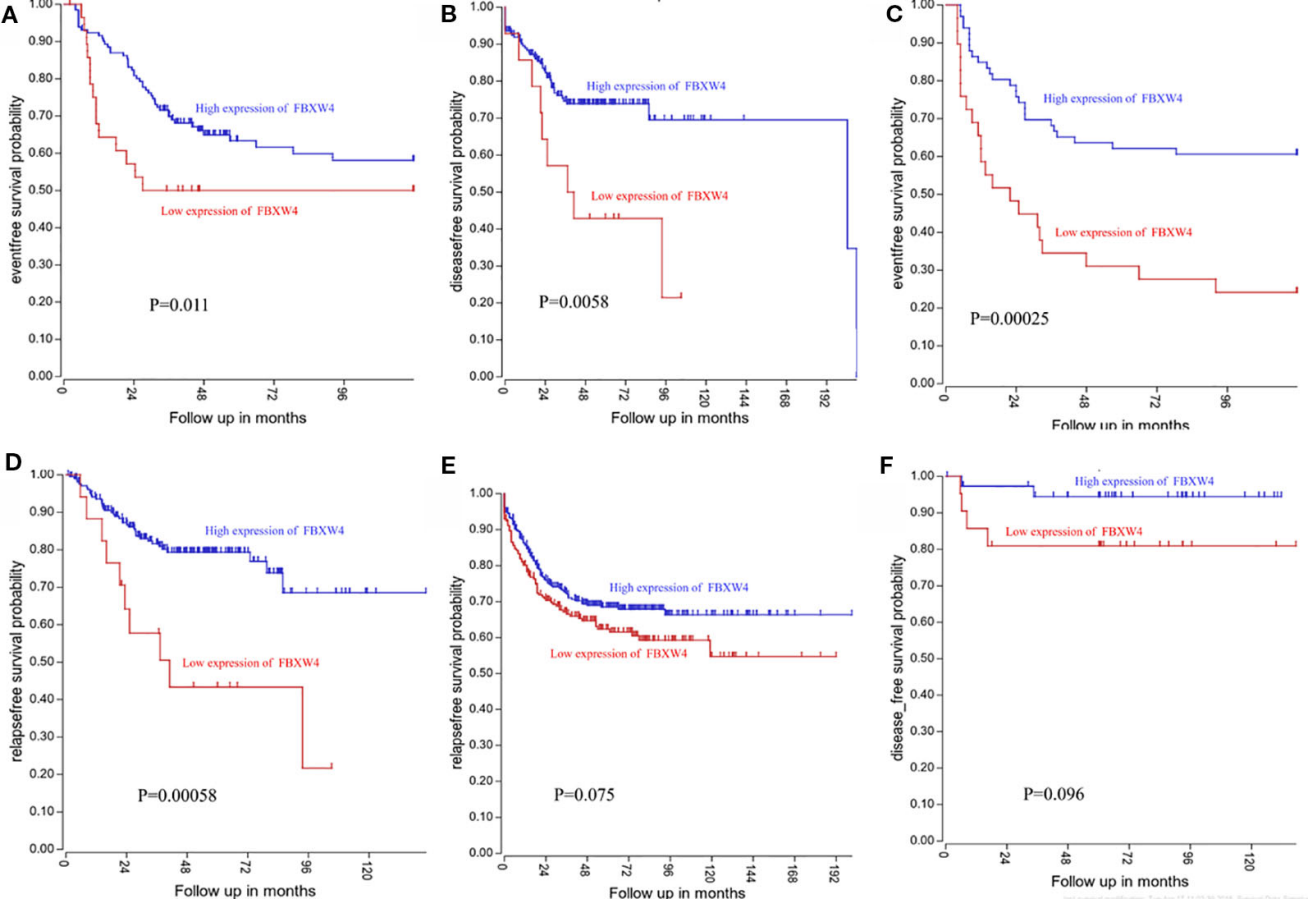
Low FBXW4 expression was correlated with a worse event-, disease-, and relapse-free survival. **(A**–**D)** Low FBXW4 expression was correlated with a significantly worse event-, disease- and relapse-free survival. (both P < 0.05). **(E)** (P = 0.075) and **(F)** (P = 0.096) Low FBXW4 expression was correlated with a worse disease- and relapse-free survival.

We analyzed the expression level of FBXW4 expression to independently validate the hub genes obtained from our data set by comparing mCRC tissues of chemotherapy-resistance and-sensitive cases from two independent data sets (our data and GSE69657). In internal testing data sets, the results indicated that the relative FBXW4 expression was significantly increased in chemotherapy-sensitive tissues compared with chemotherapy-resistance tissues (11.40 ± 0.40 vs 13.38 ± 0.60, P = 0.000; **Figure 4B**) in our data. These results were further confirmed in the external data set (GSE69657), in which the relative FBXW4 expression levels in the chemotherapy-sensitive and-resistance tissues were 231.13 ± 52.16 and 317.35 ± 102.57, respectively (P = 0.006; **Figure 4C**). The ROC curve validated that FBXW4 could efficiently distinguish chemotherapy-resistance from chemotherapy-sensitive mCRC cases in both our data set (P = 0.008, AUC = 1; **Figure 4D**) and the GSE69657 data set (P = 0.038, AUC = 0.725; **Figure 4E**). In addition, a meta-analysis of 15 GEO-sourced data sets mined from the Oncomine database showed that FBXW4 mRNA levels were significantly lower in CRC tissues than in normal colon tissues (P < 0.001; **Figure 4A**). The R2: Genomics Analysis and Visualization Platform was used to generate Kaplan-Meier overall survival curves using the “Tumor Colon-Sieber-290-MAS5.0-u133p2” data set, “Tumor Colon-Smith-232-MAS5.0—u133p2” data set, “Tumor Colon MSI-status (Core Exon)-Sveen-95-rma-sketch- huex10p” data set, and “Tumor Colon (Core-Exon)-Sveen-333-rma-sketch - huex10p data set”. Low FBXW4 expression was correlated with a significantly worse event and relapse-free survival (both P < 0.05; **Figures 5A–D**). Moreover, we found that in the “Tumor Colon (KRAS mut)-Hase-59-MAS5.0-u133p2” data set (P = 0.096), and the “Tumor Colon CIT (Combat)-Marisa-566-rma-u133p2” data set (P = 0.076) the p-value was higher than 0.05 in R2, however the high expression of FBXW4 was associated with a better event and relapse-free survival than in the lower groups (**Figures 5E, F**).

To further identify the FBXW4 expression in the FOLFOX chemotherapy CRC patients, we detected the FBXW4 expression in the chemotherapy-resistance/sensitive mCRC patients who received the FOLFOX regimen. The results demonstrated that the FBXW4 expression was lower in the drug resistance group than that in the drug sensitive group (P = 0.01, **Figures 4F, G**; **Supplemental Figure 1**). Moreover, the K-M analysis indicated that high FBXW4 expression was associated with better prognosis in mCRC patients (**Figure 4H**, **Supplemental Figure 1**).Moreover, FBXW4 expression was also detected in the FOLFOXresistance cell lines. The characteristics of the drug resistant cell lines had already been reported in our previous study (Zhang et al., 2018). The results demonstrated that the FBXW4 expression was lower in theFOLFOX resistant cell lines than in the parental cell line.

To further explore the FBXW4 expression in accepting FOLFIRI regimen mCRC patients. We analyzed the FBXW4 expression in mCRC patients who received the FOLFIRI regimen before surgery. The results demonstrated that the FBXW4 expression was similar between the FOLFIRI-resistant and -sensitive groups (P = 0.67), in the **Supplementary Figure 4**. Combined with the present studied dataset, we found that the higher expression of the FBXW4 predicted the FOLFOX sensitive in the mCRC patients. However, in the accepting FOLFIRI mCRC patients the expression of the FBXW4 was similarly in the resistance/sensitive group.

### GSEA

**Figure 6:**
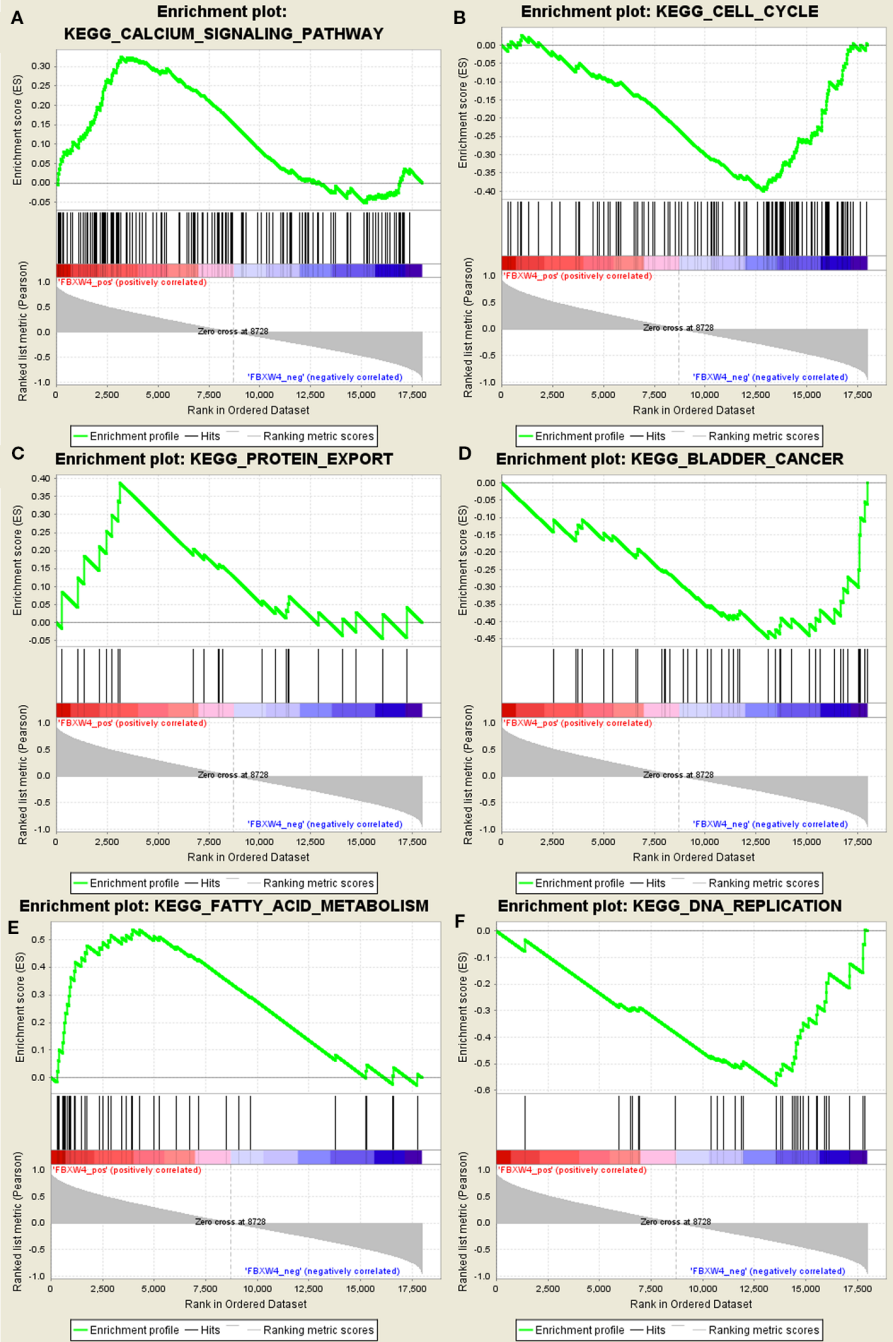
GSEA using our data. **(A)** Calcium signal pathway. **(B)** Cell cycle. **(C)** Protein export. **(D)** Bladder cancer. **(E)** Fatty acid metabolism. **(F)** DNA replication signal pathway.

GSEA was conducted to determine the potential mechanism for FBXW4 involvement in chemotherapy-resistance in CRC. Our data demonstrated that the enriched negatively correlated KEGG pathways included the DNA replication signaling pathway (**Figure 6**), the bladder cancer signaling pathway, and the cell cycle signaling pathway. The positively correlated pathways included the calcium signaling pathway, the fatty acid metabolism signaling pathway, and the protein export signaling pathway.

## Discussion

Chemotherapy-resistance to the FOLFOX/CapeOX regimen is a complex phenomenon. It remains a major problem for patients with *a priori* resistant tumors. In this study, WGCNA, an advanced methodology of multigene analysis, was performed to identify gene co-expression modules related to chemotherapy-resistance based on the similarity of the expression patterns. The black module was identified as a chemotherapy resistance hub module. Furthermore, PPI analysis demonstrated that FBXW4 was the hub gene in the black module. Moreover, to further identify the relationship between FBXW4 and chemotherapy-resistance, we analyzed the expression of FXBW4 using ROC curves in both our data and the GSE69657 data. In addition, we obtained consistent results in the R2 and Oncomine database. Moreover, the FBXW4 expression was identified in our datasets using immunohistochemical analysis.

To date, reliable molecular markers for the FOLFOX/CapeOX regimen resistance in CRC patients are still unavailable, especially for the primary resistance Moreover, we analyzed the data in the GEO dataset, and found eight datasets, GSE19860, GSE52735, GSE104645, GSE14095, GSE83889, GSE72968, GSE110785, and GSE69657 were contained CRC patients who received FOLFOX chemotherapy (Watanabe et al., 2011; Li et al., 2013; Estevez-Garcia et al., 2015; Tong et al., 2015; Del et al., 2017; Cherradi et al., 2017; Kwon et al., 2017; Okita et al., 2018; Cherradi et al., 2019). We further analyzed the above datasets, the results demonstrated that the datasets, GSE104645, GSE110785, GSE19860, GSE52735, and GSE14095, including the information about acquired resistance and lacked the primary resistance. The dataset, GSE83889, included the pathological stage III CRC patients and unable provide effective evidence of resistance to FOLFOX. The dataset GSE72968 came from European multi-center study including 26 patients and supplied the information about the primary resistance. However, we found that the primary FOLFOX-resistance dataset from non-European was still scared currently. Thus, the information about the primary resistance from Asian, non-European patients in our study, could serve as a valid non-European supplement to dataset from European. Although, in the present study we supplied the small number of samples, we supplied the information could serve as a valid non-European supplement to dataset from European.

To identify reliable primary FOLFOX-resistance biomarkers from the non-European mCRC patients, microarray expression profiling has been utilized to screen for biomarkers of chemotherapy-resistance in mCRC patients from Chinese, non-European. However, the traditional molecular biology methodology identifies differentially expressed genes and it is always difficult to examine the biological information between the genes and biological functions in each sample. WGCNA has emerged as an effective method to discover the relationship between networks/genes, phenotypes and samples to avoid the defects of the traditional method (Gao et al., 2016; Bakhtiarizadeh et al., 2018; Magani et al., 2018). In this study, we detected the mRNA expression in 11 mCRC patients who were defined as chemotherapy-resistant or -sensitive according to the RESIST criterion using microarray analysis. To identify the expression of the gene in each group that was associated with chemotherapy-resistance, we performed GO functional and KEGG pathways analysis in the differentially expressed transcripts (P < 0.05, Fold Change > 2). The result demonstrated that resistance to FOLFOX regimen in mCRC patients was associated with the immune system. Moreover, we further detected TILs expression in mCRC patients receiving chemotherapy. The results indicated that higher levels of TILs in the tumor tissue were associated with a better prognosis. In line with our results, many studies have revealed that the dysregulation of the immune system is associated with resistance to the FOLFOX regimen (Tanis et al., 2015; Limagne et al., 2016; Emile et al., 2017).

After completing our initial analyses, we performed WGCNA to identify the “real” hub gene. The results from the WGCNA demonstrated that the black module is the hub module, and that the genes in the black module were enriched for cell proliferation, the MAPK signaling pathway, and the PI3K signaling pathway, which has already been reported to be associated with resistance to multiple chemotherapy regimens in cancers. These findings were similar to observations reported in our previous study (Russo et al., 2009; Arqués et al., 2016; Ma et al., 2017; Zhang et al., 2018). These results are in accordance with our previous study (Russo et al., 2009; Arqués et al., 2016; Ma et al., 2017; Zhang et al., 2018). Thus, the black module was significantly associated with FOLFOX-resistance. As described in the previous studies (Liu et al., 2017; Wang et al., 2018), the PPI network and MOCDE were performed to screen for the “real” hub gene. These results suggested that FBXW4 had the highest correlation coefficient out of all of the genes screened using the PPI network and MOCDE. To identify whether FBXW4 was the “real” hub gene, we analyzed the expression of FBXW4 in the present data and in our previous data (GSE69657). Two independent data sets verified that FBXW4 was the most relevant gene for chemotherapy resistance and was overexpressed in chemotherapy-sensitive CRC patients' tissues compared with chemotherapy-resistant patients' tissues. The ROC curve demonstrated that FBXW4 could effectively distinguish between chemotherapy-resistant and chemotherapy-sensitive cases. To the best of our knowledge, this study was the first to implicate and verify FBXW4 as an effective new marker for chemotherapy response prediction. In addition, the FBXW4 expression was also identified in our dataset, and the results were in good accordance with the bioinformatics results. Taken together, our results indicated that FBXW4 is a protecting factor in the mCRC patients.

FBXW4 is a member of the F-box protein family, which was identified through screening proteins that bind SKP1 (Cenciarelli et al., 1999; Winston et al., 1999). Previous studies have reported that F-box proteins regulate specific substrates in diverse biological processes, including cell growth and division, cell development and differentiation, and cell survival and death (Skaar et al., 2013). FBXW4 was originally mapped as the region on human chromosome 10 that was the causal locus in the limb malformation disorder split hand and foot 3 (SHFM3) (Gurrieri et al., 1996; Raas-Rothschild et al., 1996; Ianakiev et al., 1999; Ozen et al., 1999). A previous study demonstrated that decreased FBXW4 expression correlated with poor survival of non-small cell lung cancer patients (Lockwood et al., 2013). In the present study, we further identified the relationship between FBXW4 expression and prognosis. The results revealed that downregulation of FBXW4 favored tumor relapse and that this was correlated with decreased survival in another four independent R2 online databases. In the other two datasets, we also found that patients with high expression of FBXW4 had a better prognosis than the low expression group, even though the high expression of FBXW4 did not significantly increase survival time. Additionally, we analyzed FBXW4 expression in CRC tissue by conducting a meta-analysis of 15 GEO-sourced data sets in Oncomine. The result demonstrated that the expression of FBXW4 was higher in the paracancerous tissue than cancerous tissue. Therefore, our results identified FBXW4 as a novel putative anti-oncogene and suggested that it could possibly be exploited as a better prognostic indicator in patients with CRC. The previous study reported that FBXW4 regulates the ubiquitination and cell cycle (Lockwood et al., 2013). In the present study, we found that FBXW4 was not only related to the cell cycle signaling pathway, but was also related to the DNA replication signaling pathway, which is associated with oxaliplatin resistance (Martinez-Balibrea et al., 2015; Hu et al., 2019). However, the underlying mechanism by which FBXW4 increases FOLFOX regimen sensitivity in mCRC patients is still unclear. Thus, further investigation is warranted.

There were some limitations to the current study. Firstly, the relatively small sample size was a major limitation of our study. Due to the study design, we included patients with mCRC at diagnosis and without any treatment before biopsy from colonoscopy, which limited the sample size of our study. We will continue to expand our sample size in our future studies. Secondly, the function of the involved pathways of FBXW4 were studied using GeneChip Array and bioinformatics methods. These results need to be validated using *in vitro* and *in vivo* experimental studies. Future research could include gain- and loss-of-function experiments and xenograft animal studies.

## Conclusion

In conclusion, we analyzed the mRNA expression of 11 mCRC patients from China, non-European, receiving preoperative FOLFOX chemotherapy using microarray analysis which focused on the primary resistance from Asian, non-European patients, and could serve as a valid non-European supplement to dataset from European populations Through WGCNA, we demonstrated that the crucial functions enriched in chemotherapy-resistance modules were cell proliferation, the MAPK signaling pathway, and the PI3K signaling pathway. Additionally, we identified and validated FBXW4 as a new effective predictor for chemotherapy sensitivity and a prognostic factor for survival of CRC patients. These results are of great clinical significance to screen CRC patients who are suitable for FOLFOX chemotherapy. The mechanism of FBXW4-mediated chemotherapy resistance may involve the DNA replication signaling pathway and the cell cycle. Nevertheless, more insightful molecular mechanisms may be obtained from future studies.

## Data Availability Statement

The datasets generated for this study can be found in NCBI GEO accession GSE138912.

## Ethics Statement

This study was carried out in accordance with the committee of Fujian Medical University Union Hospital with written informed consent from all subjects. All subjects gave written informed consent in accordance with the Declaration of Helsinki. The protocol was approved by committee of the Fujian Medical University Union Hospital.

## Author Contributions

YZ, LS, XW, YS, ZX, and XL designed the experiments, performed the experiments, analyzed the data, and wrote the paper. YC, MX, and PC performed the experiments. All authors read and approved the final manuscript.

## Funding

This study was supported by the National Natural Science Foundation of China (No 81472777, 81902378), Science Foundation of the Fujian Province, (No 2017J01296), National Clinical Key Specialty Construction Project (General Surgery) of China (No 2012-649), Joint Funds for the innovation of science and Technology, Fujian province (2017Y9104, 2018Y9021), and the Startup Fund for Scientific Research, Fujian Medical University (2017XQ1029, 2018QH2027).

## Conflict of Interest

The authors declare that the research was conducted in the absence of any commercial or financial relationships that could be construed as a potential conflict of interest.

## Supplementary Material

Supplemental Figure 1 Representative figures of FBXW4 expression cancerous **(A–D)** and adjacent cancerous **(E–H)** colon tissues.

Supplemental Figure 2 Representative figures of CD3+/4+/8+ expression in cancerous colon tissues. CD3+ expression **(A–D)**, CD4+ expression **(E–H)** and CD8+ expression **(I–L)**.

Supplemental Figure 3 Cutoff points for CD3+/4+/8+ expression determined by X-tile program. X-tile analysis divided the entire cohort into the training sets (shown in the upper-left quartile of Figures **A**, **D**, and **G**) and matched validation sets (shown in the bottom X-axis of Figures **A**, **D**, and **G**) based on patient survival data. The black dot in the validation set represents the exact cutoff values for the CD3+/4+/8+ expression. The entire cohort was divided into low (blue) and high (gray) CD3+/4+/8+ expression groups based on the optimal cut-off point (CD3+, 1; CD4+, 1; CD8+, 2);, as shown on a histogram of the entire cohort **(B**, **E**, and **H)**, a Kaplan-Meier curve of overall survival for CD3+ (C), CD4+ **(F)**, and CD8+ (I) for the optimal cut-off point.

Supplemental Figure 4 **(A)**The immunohistochemical score of FBXW4 in FOLFIRI-resistance and -sensitive cancerous tissue (P = 0.67). **(B)** The overall survival in mCRC patients with FBXW4 high-expression who received FOLFOX or FOLFIRI chemotherapy (P < 0.01).

## Abbreviations

WGCNA, weighted gene co-expression network analysis; CRC, colorectal cancer; GEO, Gene Expression Omnibus; STRING, Search Tool for the Retrieval of Interacting Genes; PPI, protein-protein interaction; GSEA, Gene set enrichment analysis; ROC, receiver operating characteristic curve; AUC, area under the curve.
