# Supplementary figures and images for: FBXW4 Acts as a Protector of FOLFOX-Based Chemotherapy in Metastatic Colorectal Cancer Identified by Co-Expression Network Analysis
Source: Front Genet. 2020 Mar 11;11:113. doi: 10.3389/fgene.2020.00113 (PMC7078371; doi:10.3389/fgene.2020.00113)

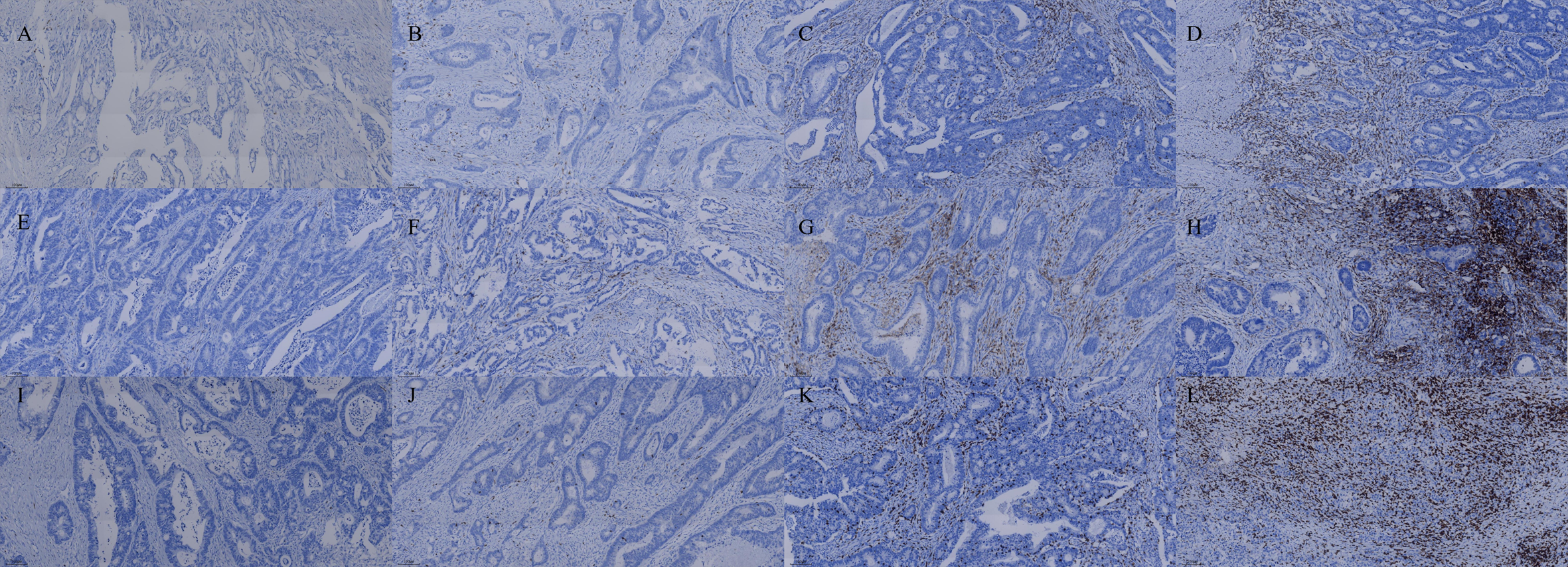

Supplement: Supplemental Figure 1 — Representative figures of FBXW4 expression cancerous (A–D) and adjacent cancerous (E–H) colon tissues. [file Image_1.tif]

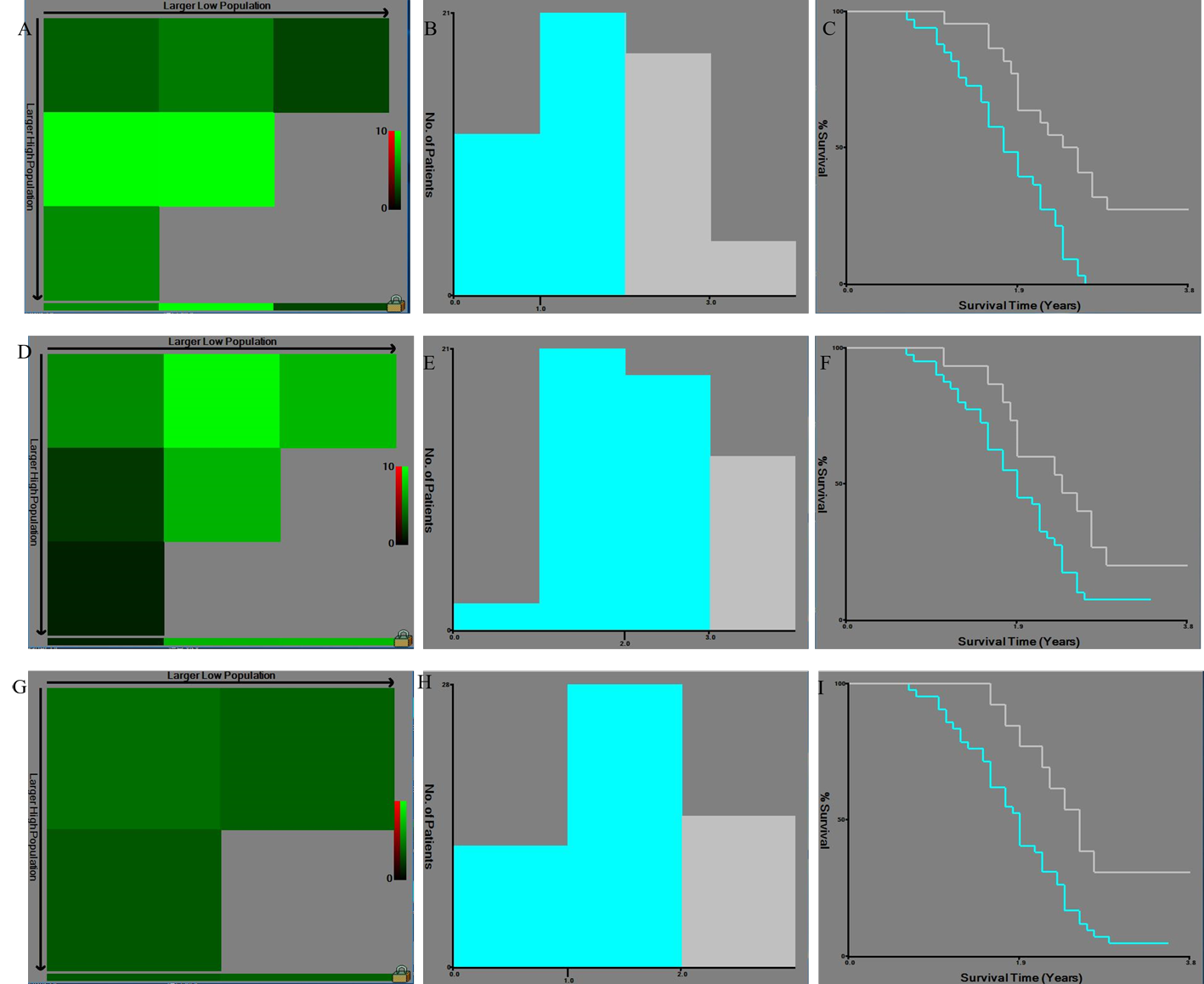

Supplement: Supplemental Figure 2 — Representative figures of CD3+/4+/8+ expression in cancerous colon tissues. CD3+ expression (A–D), CD4+ expression (E–H) and CD8+ expression (I–L). [file Image_2.tif]

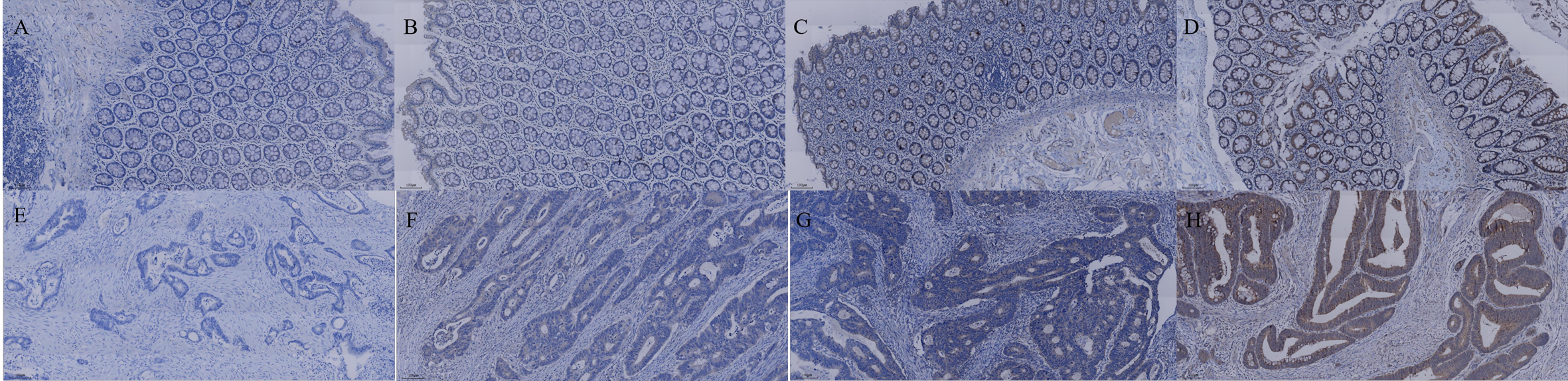

Supplement: Supplemental Figure 3 — Cutoff points for CD3+/4+/8+ expression determined by X-tile program. X-tile analysis divided the entire cohort into the training sets (shown in the upper-left quartile of Figures A, D, and G) and matched validation sets (shown in the bottom X-axis of Figures A, D, and G) based on patient survival data. The black dot in the validation set represents the exact cutoff values for the CD3+/4+/8+ expression. The entire cohort was divided into low (blue) and high (gray) CD3+/4+/8+ expression groups based on the optimal cut-off point (CD3+, 1; CD4+, 1; CD8+, 2);, as shown on a histogram of the entire cohort (B, E, and H), a Kaplan-Meier curve of overall survival for CD3+ (C), CD4+ (F), and CD8+ (I) for the optimal cut-off point. [file Image_3.tif]

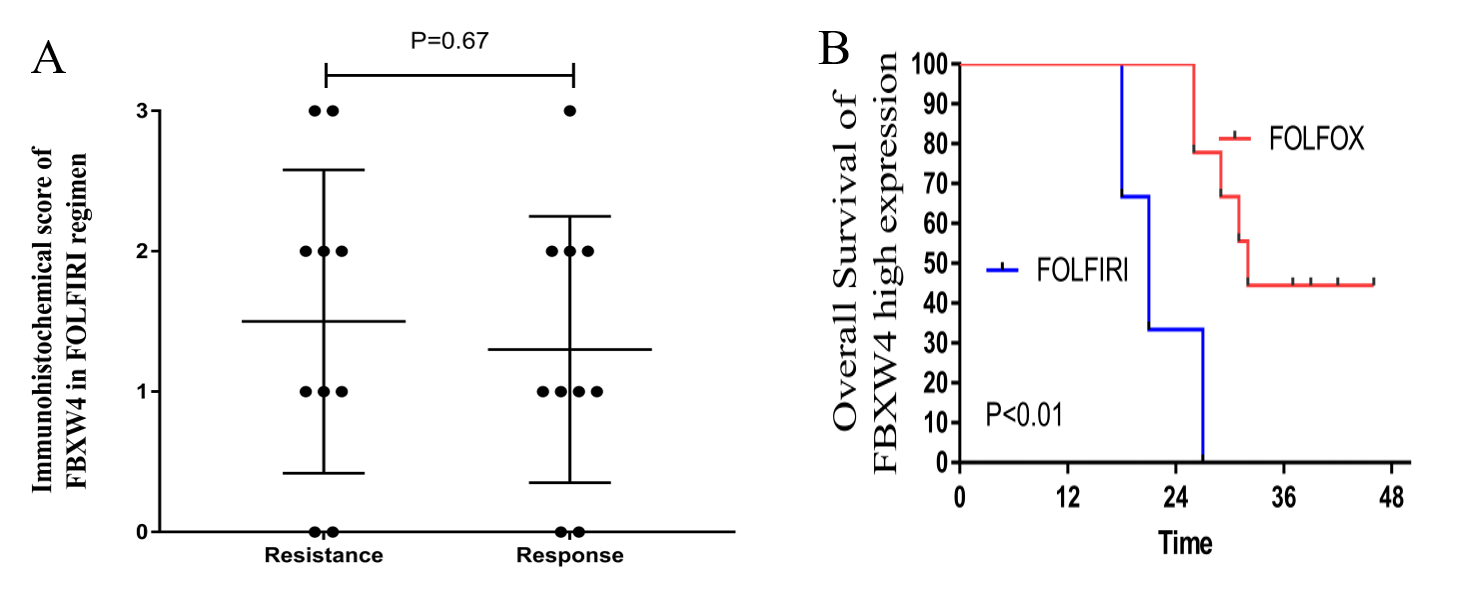

Supplement: Supplemental Figure 4 — (A)The immunohistochemical score of FBXW4 in FOLFIRI-resistance and -sensitive cancerous tissue (P = 0.67). (B) The overall survival in mCRC patients with FBXW4 high-expression who received FOLFOX or FOLFIRI chemotherapy (P < 0.01). [file Image_4.tif]
