# Supplementary material for: FBXW4 Acts as a Protector of FOLFOX-Based Chemotherapy in Metastatic Colorectal Cancer Identified by Co-Expression Network Analysis
Source: Front Genet. 2020 Mar 11;11:113. doi: 10.3389/fgene.2020.00113 (PMC7078371; doi:10.3389/fgene.2020.00113)
Supplement: Supplementary file 5 [file Table_1.docx]

**Supple Table 1** Clinicopathological details of 11 mCRC patients

| **No** | **Age（yrs）** | **Sex** | **Initial metastasis** | **Tumor location** | **Chemotherapy regimen** | **Tumor response** | **Pathology stage** |
| --- | --- | --- | --- | --- | --- | --- | --- |
| WYY | 65 | F | Liver | rectal | FOLFOX | PD | T4N2M1 |
| CHP | 67 | M | Liver | Sigmoid | FOLFOX | PR | T3N0M1 |
| LYH | 79 | M | Liver | Ascending colon | FOLFOX | PR | T3N0M1 |
| CYY | 61 | F | Liver | rectal | FOLFOX | PR | T3N1M1 |
| CLJ | 59 | M | Liver | rectal | FOLFOX | SD | T3N1M1 |
| CBX | 74 | F | Liver | Sigmoid | FOLFOX | PR | T3N1M1 |
| LZX | 65 | F | Liver | Ascending colon | FOLFOX | SD | T3N2M1 |
| ZJM | 47 | F | Liver | rectal | FOLFOX | PD | T4N1M1 |
| ZXH | 29 | M | Liver | rectal | FOLFOX | SD | T4N2M1 |
| YCD | 45 | M | Liver | rectal | FOLFOX | PD | T3N2M1 |
| HGZ | 74 | F | Liver | rectal | FOLFOX | SD | T3N1M1 |

**M**, male; **F**, female; **FOLFOX**, 5-fluorouracil/folinic acid plus oxaliplatin; **PR**, partial responses; **SD**, stable disease; **PD**, disease progression
